# Bayesian Optimization of insect trap distribution for pest monitoring efficiency in agroecosystems

**DOI:** 10.3389/finsc.2024.1509942

**Published:** 2025-01-22

**Authors:** Eric Yanchenko, Thomas M. Chappell, Anders S. Huseth

**Affiliations:** ^1^ Global Connectivity Program, Akita International University, Akita, Japan; ^2^ Department of Plant Pathology and Microbiology, Texas A&M University, College, Station, TX, United States; ^3^ Department of Entomology and Plant Pathology and North Carolina Plant Science Initiative, North Carolina State University, Raleigh, NC, United States

**Keywords:** *Helicoverpa zea*, sampling efficiency, adaptive sampling, eco-efficiency, integrated pest management

## Abstract

Insect trap networks targeting agricultural pests are commonplace but seldom optimized to improve precision or efficiency. Trap site selection is often driven by user convenience or predetermined trap densities relative to sensitive host crop abundance in the landscape. Monitoring for invasive pests often requires expedient decisions based on dispersal potential and ecology to inform trap placement. Optimization of trap networks using contemporary analytical approaches can help users determine the distribution of traps as information accumulates and priorities change. In this study, a Bayesian optimization (BO) algorithm was used to learn more about the optimal distribution of a fine-scale trap network targeting *Helicoverpa zea* (Boddie), a significant agricultural pest across North America. Four years of pheromone trap monitoring was conducted at the same 21 locations distributed across ~7,000 square kilometers in a five-county area in North Carolina, USA. Three years of data were used to train a BO model with a fourth year designated for testing. For any quantity of trap locations, the approach identified those that provide the most information, allowing optimization of trapping efficiency given either a constraint on the number of locations, or a set precision required for pest density estimation. Results suggest that BO is a powerful approach to enable optimized trap placement decisions by practitioners given finite resources and time.

## 1 Introduction

Efficient management of agricultural pest insects requires accurate and timely information about the relative abundance of populations from plant to landscape scales. Understanding where populations occur is also important because many insect pests have uneven distributions within fields and across agroecosystems. For decades, Integrated Pest Management (IPM) practitioners have used pest trap networks as one tool to monitor pest activity and inform growers about infestation risk during the growing season. Pest density estimation using traps informs grower management decisions when used in combination with traditional crop scouting, but collecting this information is costly and rarely optimized for specific cropping systems. Examples of management thresholds using traps exist, but as pest management practices change or monitoring tools evolve (e.g., trap technology, pheromone blend), the relevance of these decision tools decreases. Improving analytical approaches through contemporary modeling will make trap networks more adaptable to enable better management decisions. This improvement addresses a central principle of IPM, which seeks to maximize sustainability of pest management through diversified management practices in the context of agroecosystems (1).

A central challenge to the continued refinement and adoption of trap network information arises from the translation of pest activity to direct negative impacts on crops. Recent literature has explored trap network optimization for agricultural or medical pest populations in simulated landscapes that include parameters to include varying ecological complexity and stakeholder goals (2–6). A general theme of this work is that pest populations can be viewed as processes and monitored using simulation techniques developed to detect changes or abnormalities. However, pest populations in agroecosystems are often aggregated in space in addition to being temporally dynamic. Contagion is also a feature of wild insect populations that can challenge assumptions involved in risk assessment. This results in a need to monitor pest populations through time, but it also necessitates attention to the spatial distribution of trap network sites. Techniques useful for generating expectations of pest densities at unsampled geospatial locations exist. For example, Gaussian process regression or Kriging approaches can be useful to combine spatial with temporal aspects of sampling optimization and may increase efficacy of trap networks if users are willing to reconfigure distributions based on stakeholder goals (i.e., detection of large populations). Although these studies can accommodate complex simulations and multiple objectives, they rarely use the abundance of observational data being collected in real agricultural systems to better understand how the efficiency of existing networks can be improved.

Here we develop an application of Bayesian Optimization (BO) (7) to select from a finite number of trapping locations involved in monitoring an agricultural pest lepidopteran insect, *Helicoverpa zea* Boddie. Bayesian Optimization has become a popular tool for optimizing complicated objective functions and has found success in many domains, particularly environmental monitoring and sensor selection. Practical examples of Bayesian Optimization approaches include selecting the optimal weather sensors from a predefined set to predict the maximum rainfall at unobserved locations (8), selecting sensors to monitor ozone concentrations (9), and selecting sensors for temperature monitoring in a given area (10). In our study, there preexists a certain number of locations from which adult *H. zea* abundance data have been collected using pheromone-baited Texas Hartstack traps (11). We hypothesize that the information yield of different trap locations varies and the number of traps in a network can be optimized to a smaller subset of high-value locations. In other words, for any predetermined precision requirement, trapping effort can be minimized, and for any predetermined amount of available effort, precision can be maximized. Through this application, we optimize the subset of locations at which to trap to maximize information return (coverage, precision) while minimizing costs. Important additional benefits arise from this approach, for example improvements to the expedient distribution of traps when monitoring for novel or invasive pests, or to the identification of geospatial areas or place-time combinations that are inconsistent with their surroundings in ways that expedite research into the system’s biology.

Bayesian Optimization is used to address a combinatorial problem in which the goal is to choose an optimal K locations from a possible set L , meaning there are ${\;}_{L}C_{K}$ possible sets of locations that can be chosen. Problems in combinatorics inherently require efficiency to address because of the expansive search spaces they present to brute force approaches: a realistic example of 50 potential locations from which to choose a subset of 20 to receive traps results in more than 10^13^ combinations, which is not practical to search, even if the datum at each trap site is simple and affordable to obtain. However, the necessity of efficiency is increased in this use case because the quantity to be optimized is complicated and expensive to calculate. Bayesian Optimization is thus a well-suited approach to this problem, because it can approximate a complicated loss function with a simple statistical model. In our approach, a model is fit with Bayesian regression and then maximized with an acquisition function. We evaluate the original loss function with the optimum from the surrogate model, add it to our data set, and then refit the model. A relatively simple surrogate model is more efficient to optimize in comparison to the complicated original objective function.

## 2 Materials and methods

### 2.1 Pest ecology and trap network

In the southeastern U.S., *H. zea* completes several generations that cycle through non-crop weeds and then crops that include corn (*Zea mays* L.), upland cotton (*Gossypium hirsutum* L.), soybean (*Glycine max* (L.) Merr.), and several other minor crops (12–15). Larvae are the economically important life stage because they consume vegetative and reproductive plant structures that result in crop yield loss (e.g., cotton bolls, soybean pods, and corn kernels). As the season progresses, populations grow when high-quality crop hosts become abundant in the landscape, with some of the largest seasonal populations originating from corn (15). The adult flight originating from corn often coincides with blooming cotton and soybean in early August.

To provide an early warning system for *H. zea* flights, university extension researchers maintain trap networks to monitor *H. zea* adults across the region (16). Resulting trap data is used as an early warning system to incentivize intensive in-field *H. zea* egg and larval monitoring. Importantly, growers often infer *H. zea* activity for their locations based on a small number of traps distributed across large geographic regions. For example, the North Carolina trap network has included 22 unique black light trap locations in 2024 that span approximately 36,000 km^2^ of land area from central to eastern North Carolina. Traps in this network are concentrated in major agricultural regions with the goal of providing growers with timely pest activity information (data available at: https://www.ces.ncsu.edu/trap-data/). These networks provide useful *H. zea* activity information each year but may not account for population variation across spatially and temporally heterogenous agricultural landscapes (17, 18).

**Figure 1:**
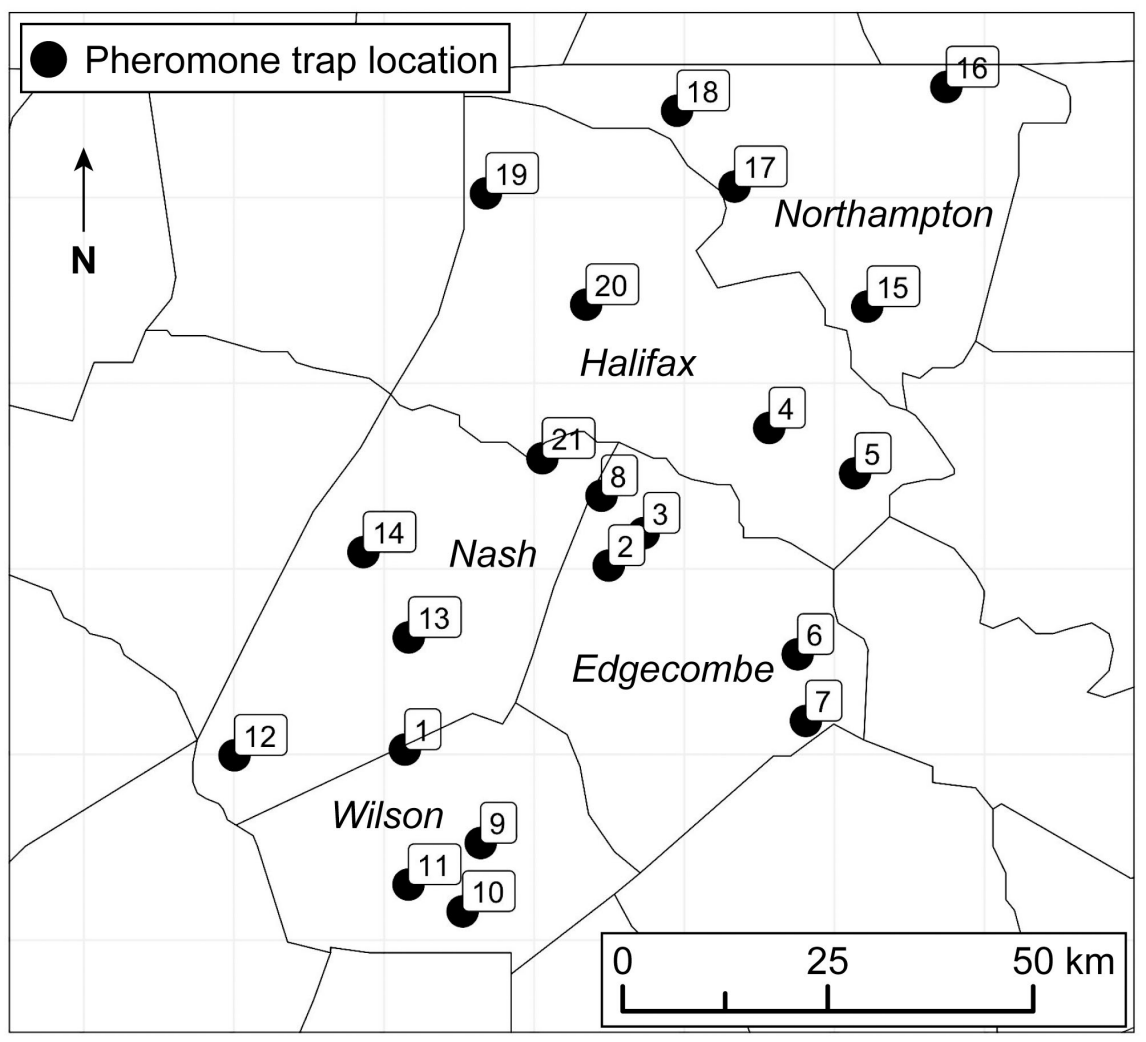
Sampling locations distributed across an intensive row crop production landscape in five North Carolina counties. Numbers correspond to trap ID used in **Figure 6**.

This study documented variation in *H. zea* adult activity in an experimental trap network distributed across 21 locations in a major row crop production landscape from 2020-2023. The trap network spans approximately 700 km^2^ of agricultural land in five North Carolina counties (i.e. Northampton, Halifax, Nash, Edgecombe, and Wilson). The fine-scale trap network monitored adult male *H. zea* using commercially available pheromone lures (PHEROCON^®^ CEW, Trécé Inc., Adair, OK) and fabricated metal Texas Hartstack traps (11). We collaborated with row crop growers to select 21 trap locations that were distributed across the study extent and located in open areas adjacent to agricultural fields. Each year, traps were installed at the same location late in July and monitored weekly for 6-11 weeks during the peak flight period (**Figure 1**). Captured moths from individual traps were returned to the laboratory and counted each week.

### 2.2 Trap data

Our dataset includes n=705 observations, L=21 locations, T=4 years (2020-2023). We have 83 site-year combinations (site 3 did not participate in 2020) and 6 - 11 weeks of observations per year. In 2020 there were 6 weeks of trapping, 2021 had 7 weeks, 2022 had 10 weeks, and 2023 had 11 weeks. In 2023, Trap 13 was missing an observation for week 2, so for this observation we imputed the average value of all other sites for this same week. Let $S=\left\{s_{1},\ldots ,s_{L}\right\}$ be the set of all sampling locations. Our response variable of interest is the cumulative pest count for each week. In particular, if $Z_{tsx}$ is the pest count (in hundreds of pests) for location s in year t at week x , then our response variable, $Y_{tsx}$ , is the *cumulative* pest count for location s in year t at week x , i.e.,


$$Y_{tsx}=\sum_{j=1}^{x}Z_{tsj}$$

Note that by definition, $Y_{t,s,x}\leq Y_{t,s,x+1}$ .

### 2.3 Logistic growth model

The logistic growth curve represents biological population growth by including both exponential increase and a density-limiting carrying capacity. It is defined as


(1) $$f\left(x\right)=\frac{\beta }{1+exp\left\{-\left(x-\gamma \right)/k\right\}}$$

for all x , where x represents the week of the observation. The model is defined by three parameters. β is supremum of the function, and here represents the maximum number of cumulative pests. A larger value of β means a greater number of pests. The growth parameter, k , represents how quickly the pest population is increasing, and is inversely correlated with growth rate. If k is large, then the growth is slower, while a small k indicates fast growth. Finally, γ is the midpoint and represents where the function attains half of its supremum, i.e., f(γ)=β/2 .  A larger γ means that the midpoint is later in the growing season, and vice-versa for a smaller γ .

As formulated in Equation 1, the logistic growth curve is the same at each trapping location. Because spatial dependencies among trapping locations are expected, β and γ depend on location. Mathematically, the model becomes


(2) $$Y_{tsx}\sim Normal\left(\mu \left(x;s\right),\sigma^{2}\right)$$

where


$$\mu \left(x;s\right)=\frac{\beta \left(s\right)}{1+exp\left[-\left\{x-\gamma \left(s\right)\right\}/k\right]}$$

The asymptote parameter, β , has been replaced with a spatially-varying parameter, β(s) . Similarly, the midpoint γ , is now modeled spatially with γ(s) . Spatial terms are modeled as a Gaussian Process with exponential correlation, i.e.,


$$\beta \left(s_{1}\right),\ldots ,\beta \left(s_{L}\right)\sim Normal\left(0,\sigma_{\beta^{2}}\Sigma_{\beta }\right)$$

and

$\gamma \left(s_{1}\right),\ldots ,\gamma \left(s_{L}\right)\sim Normal\left(0,\;\sigma_{\gamma^{2}}\;\Sigma_{\gamma }\right)$ ,

where $\Sigma_{\beta }$ and $\Sigma_{\gamma }$ represent exponential correlation structures, a special case of Matern correlation. If Σ is an exponential correlation matrix, then


$$\Sigma_{ij}=exp\left(-\left|\left|s_{i}-s_{j}\right|\right|/\rho \right)$$

where ρ is the spatial range parameter, and ||⋅|| is the Euclidean distance between trapping locations. In this correlation function, points are more correlated if they are closer together, and the correlation decreases exponentially fast with distance. This model was then fit using all locations and all years (before 2023) via Bayesian inference. Please see the **Supplementary Materials** for full prior specification.

There are several noteworthy points about this model. First, the model has spatial variation, but is the same for each year, i.e., there is no temporal component. There were not enough years of data to accurately learn a parameter differentiating years, and the model thus overfit on year when such a parameter was included. Next, the model has spatial variation in the supremum and midpoint, but not in the growth parameter. Additional models were tested with spatial variation in k , but there was very little variation among sites. Thus, the growth parameter was fixed across space. Additionally, both β(s) and γ(s) have exponential correlation structures, but are allowed to have different spatial range parameters, $\rho_{\beta }$ and $\rho_{\gamma }$ . Finally, covariates were not included in the model (e.g., landscape composition), because we found that models including candidate covariates tended to overfit to the data and did not provide good out-of-sample prediction. While we might reasonably assume that covariates such as landscape composition weather, etc. do affect the pest population, the current model implicitly accounts for such factors in the spatial processes. Moreover, the focus of this paper is selecting the optimal trapping locations, not necessarily the reason that these sites were chosen. We consider this latter question an important avenue for future work.

### 2.4 Optimal sampling locations

After empirically establishing the model form, we proceeded to determine how to select the optimal trapping location sets. We seek a small number of trapping locations, K , with K<L , such that if we fit a model on these K locations, then the model can most accurately predict at the L−K unobserved sites. For now, we consider K as known and fixed but we will generalize this in the experimental results.

We seek to minimize the mean log error (MLE) between the real and predicted cumulative counts at the unobserved locations in 2023. Let $S_{i}=\left\{s_{i_{1}},\ldots ,s_{i_{K}}\right\}$ be a set of K locations where $S_{i}\subseteq S$ , and let ${\hat{Y}}_{\tau sx}\left(S_{i}\right)$ be the predicted value at site $s\notin S_{i}$ in year τ=2023 for week x∈{1,…,11} , using the locations in $S_{i}$ to fit the logistic growth model. Thus, ${\hat{Y}}_{\tau sx}\left(S_{i}\right)$ are out-of-sample predictions. Then our loss function is the MLE on the sites we did not train on for 2023, defined as


(3) $$L\left(S_{i}\right)=\frac{1}{11\left(L-K\right)}\sum_{s\notin S_{i}}\sum_{x=1}^{11}log\left(\left|Y_{\tau sx}-{\hat{Y}}_{\tau sx}\left(S_{i}\right)\right|+1\right)$$

where log() is the natural logarithm. Optimization thus seeks


(4) $$\tilde{S}=arg\underset{S_{i},\left|S_{i}\right|=K}{min}L\left(S_{i}\right)$$

In this case, the optimum is the set of sampling locations, $\tilde{S}$ , such that, if the model is trained on this set of locations from 2020-2023, and then used to predict trap data from other locations in 2023, MLE will be minimized for predictions of these out-of-sample points. 2023 was chosen as the testing data subset to simulate forecasting of future data using past data. MLE was chosen for our loss function due to the following observation. The observed cumulative counts vary in orders of magnitude, i.e., the first week may have tens of counts while the cumulative sum at the end of the season can be in the thousands. By taking the logarithm of the difference between the observed and fitted values, it ensures that all errors are on the same scale, such that each component of the sum in (3) contributes approximately equally to the loss function. Please see the **Supplementary Materials** for further discussion on the loss-function as well as potential other options.

### 2.5 Bayesian optimization

**Figure 2:**
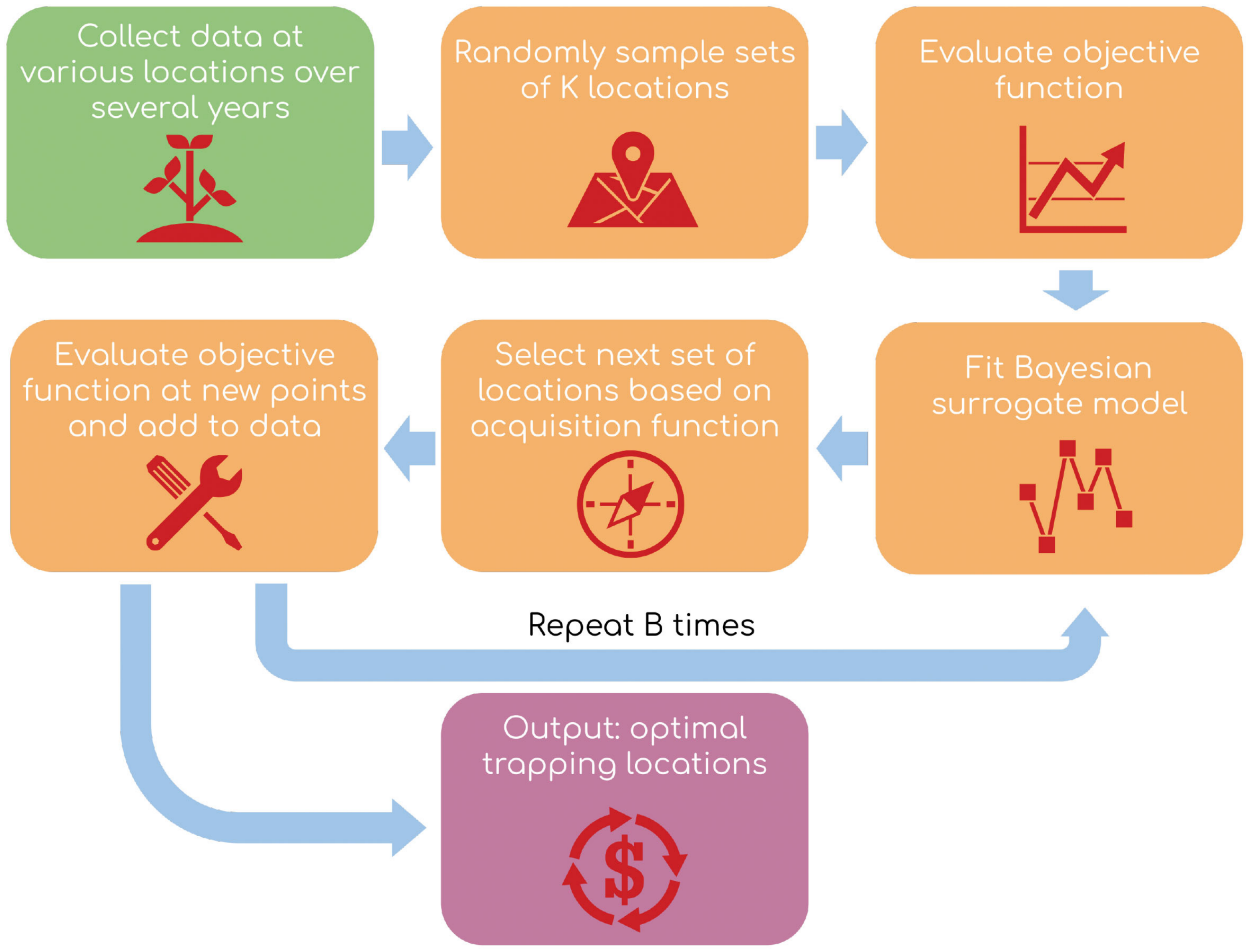
Overview of data pipeline and algorithm structure.

The loss function in Equation 3 is complex enough to complicate optimization in practice. Indeed, it is not easy to minimize this loss function directly, primarily because it is a combinatorial optimization problem with L choose K many solutions. Because fitting the logistic growth curve model takes a nontrivial amount of time, a brute force approach is not feasible. Thus, we employ Bayesian Optimization (BO) to approximate the optimal trapping locations $\tilde{S}$ . There are three main steps to a BO schema: the objective function, statistical model, and acquisition function, after Yanchenko (19). Please see **Figure 2** for an overview of the modeling workflow.

#### 2.5.1 Objective function

The first step is to evaluate the objective function at some initial points. While it is too costly to evaluate the loss function at every possible sampling set $S_{i}$ , we can evaluate it at $N_{0}$ points in a reasonable amount of time, if $N_{0}$ is small. Let $x_{i}\in {\left\{0,1\right\}}^{L}$ be such that $x_{ij}=1$ if $s_{j}\in S_{i}$ , and 0 otherwise. Since we can only choose K locations, we impose the constraint that $\sum_{j=1}^{L}x_{ij}=K$ .

Then we define $f\left(x_{i}\right)$ as the MSE for all sites $s_{j}\notin S_{i}$ and for years 2020-2022, i.e.,


(5) $$f\left(x_{i}\right)=\frac{1}{\tilde{n}}\sum_{t=2020}^{2022}\sum_{s\notin S_{i}}\sum_{x}log\left(\left|Y_{\tau sx}-{\hat{Y}}_{\tau sx}\left(S_{i}\right)\right|+1\right)$$

Where $\tilde{n}$ is the number of terms in the sum. We stress that our goal is the sampling locations $\tilde{S}$ which minimize the MSE at all locations $s\notin \tilde{S}$ for the year 2023. To train the BO algorithm, however, we cannot use the 2023 data, so our choice of optimal sampling points can only depend on the data before 2023. For our initial evaluation, we randomly sample K locations to construct each $x_{i}$ , and define $X=(x_{1}^{T},\ldots ,x_{N_{0}}^{T}{)}^{T}\in {\left\{0,1\right\}}^{N_{0}\times L}$ . For each $x_{i}$ , we fit the logistic growth model in Equation 2 and evaluate the loss function, $f\left(x_{i}\right),$ using Equation 5.

#### 2.5.2 Statistical model

Next, we need a model for the objective function in Equation 5. Let $f_{b}\left(x_{i}\right)$ be a surrogate model for $f\left(x_{i}\right)$ parameterized by ***b***. We assume that the surrogate model is linear in its parameters,


(6) $$f_{b}\left(x_{i}\right)=\sum_{j=1}^{L}x_{ij}b_{j}=x_{i}b$$

for $b={\left(b_{1},\ldots ,b_{L}\right)}^{T}$ . Since L is small, we use a Bayesian multiple linear regression to fit the model in Equation 6 with standard non-informative priors. The coefficient $b_{j}$ represents the marginal contribution of site $s_{j}$ to the overall MLE. So if $b_{j}$ is large and positive, including $s_{j}$ in $S_{i}$ will likely lead to a large MLE on our hold-out predictions. Conversely, if $b_{j}$ is negative, then the MLE will be smaller when $s_{j}$ is used to train the model. The interpretation of $b_{j}-b_{j'}$ is the increase in MLE if we used site j to train the model instead of site j' , with all else being equal. In this sense, the coefficient $b_{j}$ yields a sense of the relative increase/decrease to the testing MLE if site $s_{j}$ is used to train the model.

#### 2.5.3 Acquisition function

Lastly, we need to optimize our surrogate model $f_{b}\left(\cdot \right)$ . Recall that each coefficient $b_{j}$ represents the marginal contribution to the MSE when site $s_{j}$ is used to train the model. So, if $b_{j}$ is large in absolute value and negative, we can expect that training on this site will lead to a lower MSE. Therefore, selecting the K sites with lowest $b_{j}$ will minimize $f_{b}\left(\cdot \right)$ . For given $\hat{b}$ , the optimal sampling locations are $\tilde{x}=arg\underset{x}{min}f_{\hat{b}}\left(x\right)$ where $\tilde{x_{j}}=1$ if ${\hat{b}}_{j}\leq {\hat{b}}_{\left\{\left(K\right)\right\}}$ where ${\hat{b}}_{\left(K\right)}$ is the Kth order statistic of ${\hat{b}}_{1},\ldots ,{\hat{b}}_{L}$ . After solving for $\tilde{x}$ , we evaluate $f\left(\tilde{x}\right)$ and then re-fit (6) by appending $\tilde{x}$ and $f\left(\tilde{x}\right)$ to X and $f\left(x_{1}\right),\ldots ,f\left(x_{N_{0}}\right)$ , respectively. This step is repeated B times, and the optimal seed set is $x*=arg\underset{x\in \left\{x_{1},\ldots ,x_{N_{0}+B}\right\}}{min}f\left(x\right)$ , where the minimum is found over the $N_{0}+B$ sampled points. Then x* corresponds to our approximate optimum of (3) and therefore (5) as well.

### 2.6 Experiment

We now apply the BO method from Section 2.4 to our *H. zea* trap data. For a fixed K , we follow the method outline in Section 2.4 to obtain our approximate optimum x* . We then use the trap locations S* corresponding to x* to fit a logistic growth model based on the data from 2023 and compute the MLE on the hold-out sites for 2023, i.e. L(S*) . We do this for K=5,6,…,14 . For comparison, we also randomly select K trap locations and compute the out-of-sample MSE for 2023, which serves as a baseline method. Both the BO algorithm and random sampling are repeated for 50 Monte Carlo (MC) samples, and the average MLE is reported. We use the standard Kriging estimate to predict cumulative counts at new locations, and all Bayesian models are fit using Stan in R (20).

## 3 Results and discussion

### 3.1 MLE results

**Figure 3:**
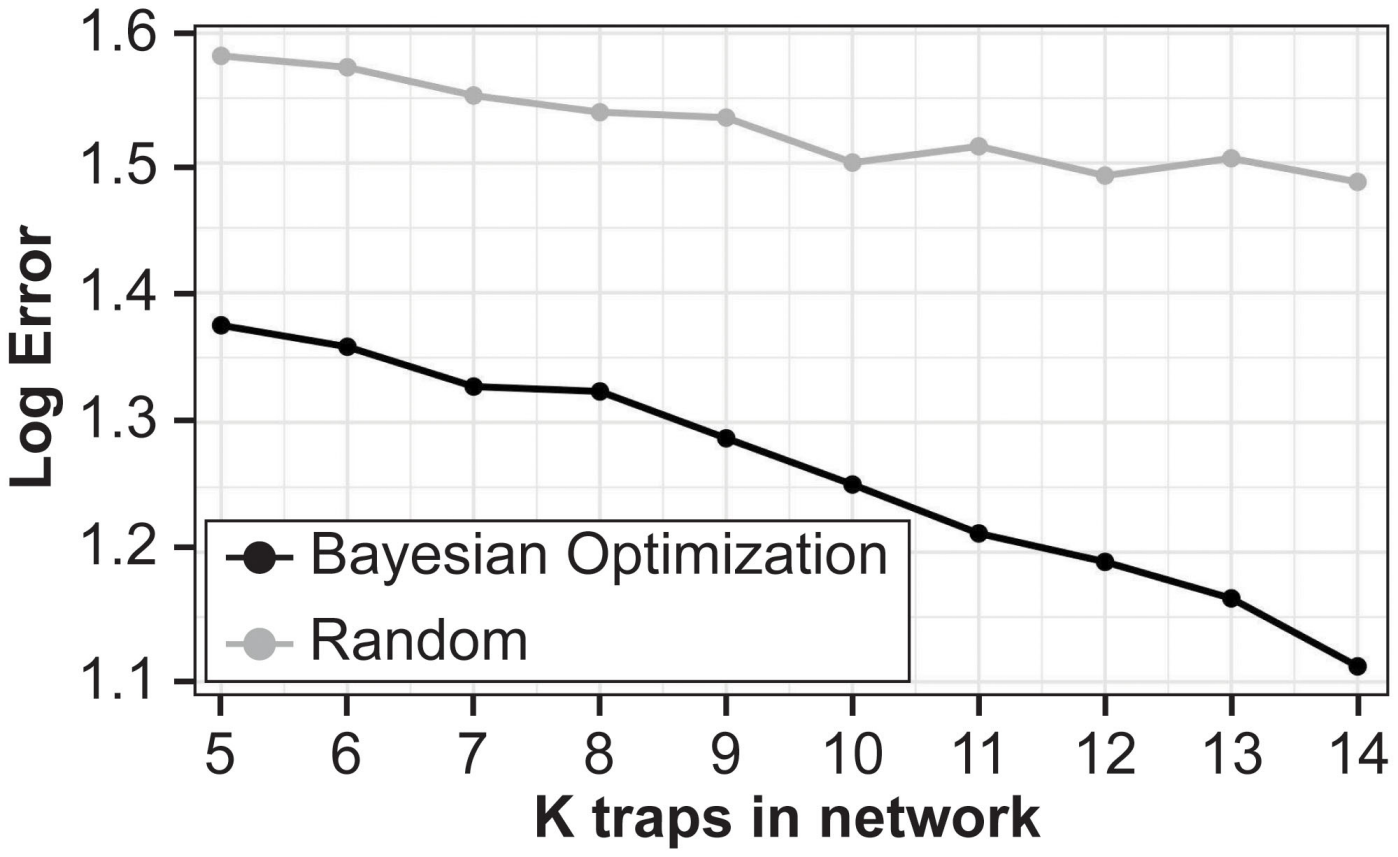
Mean log error for hold-out 2023 locations for Bayesian Optimization (black line) and random sampling locations (gray line) against number of training sites. Averaged over 50 Monte Carlo samples.

**Figure 4:**
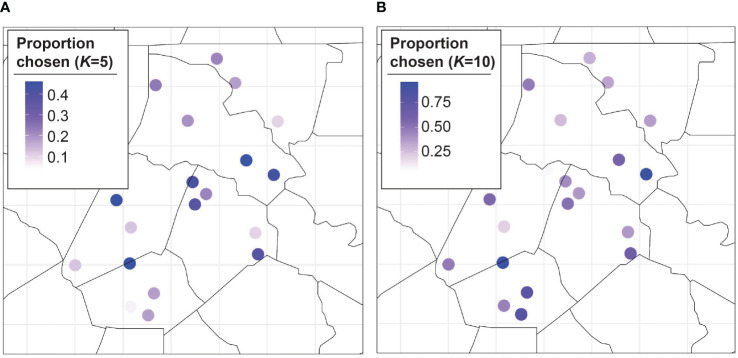
Proportion of Monte Carlo samples a trap was chosen in the optimal set for K=5 **(A)** and K=10 **(B)**.

The main results show that the proposed BO approach greatly outperforms randomly sampling locations with a lower MLE for all K (**Figure 3**). Additionally, the MLE monotonically decreases as the number of training sites increases for the BO algorithm. We plot the proportion of MC samples for which a trap location was chosen in the optimal set for K=5,10 (**Figures 4A, B**), we also plot the proportion of samples a trap was chosen in the optimal set averaged over all K (**Figure 4**). The central traps are chosen for the optimal set more often when K=5 , whereas the spread of optimal sites for K=10 is biased toward the south. When the results are averaged over all K , the southern and central locations have a higher proportion of MC samples for which they are in the optimal set.

**Figure 5:**
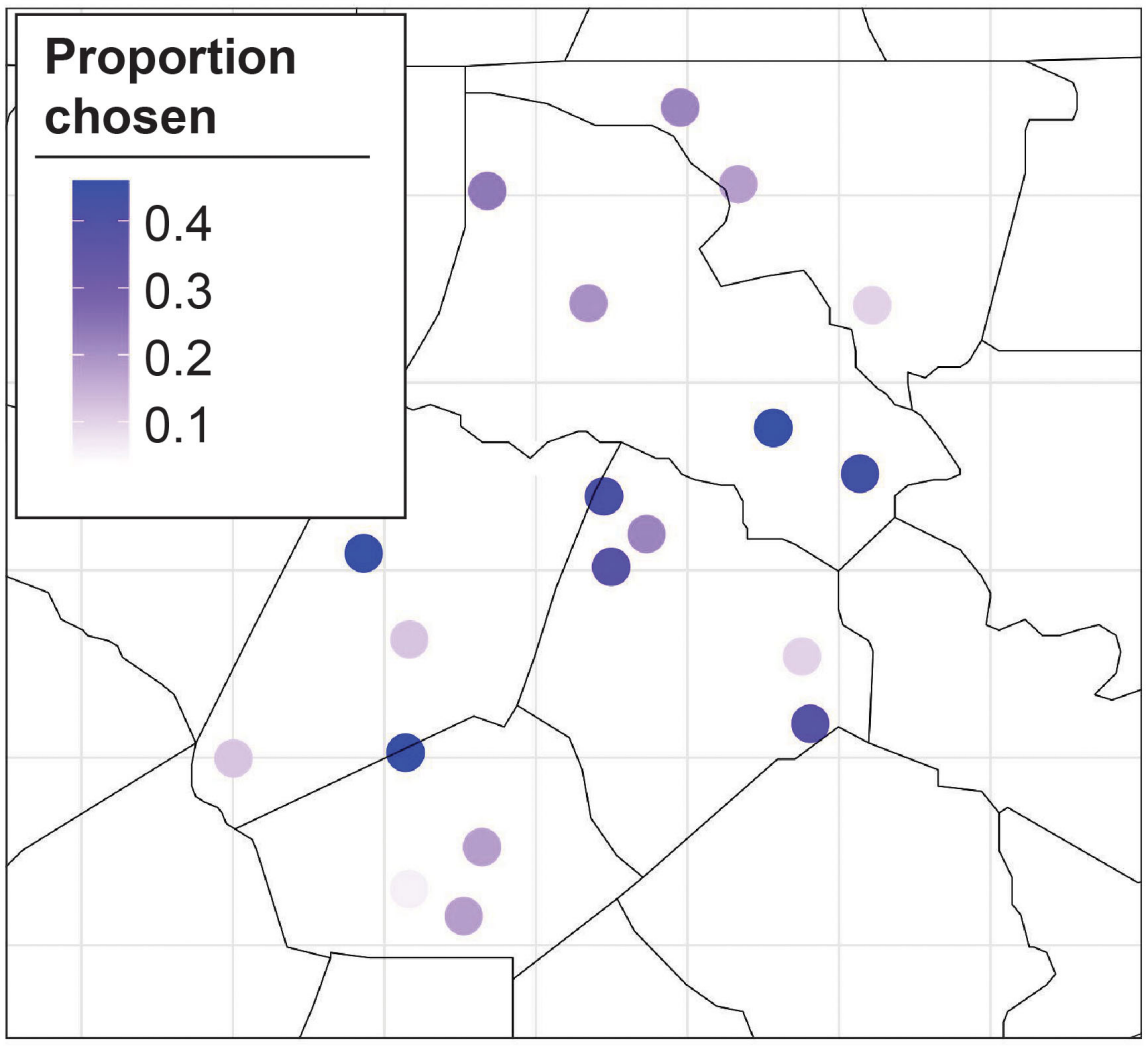
Proportion of times a site was chosen in the optimal set averaged over all K .

**Figure 6:**
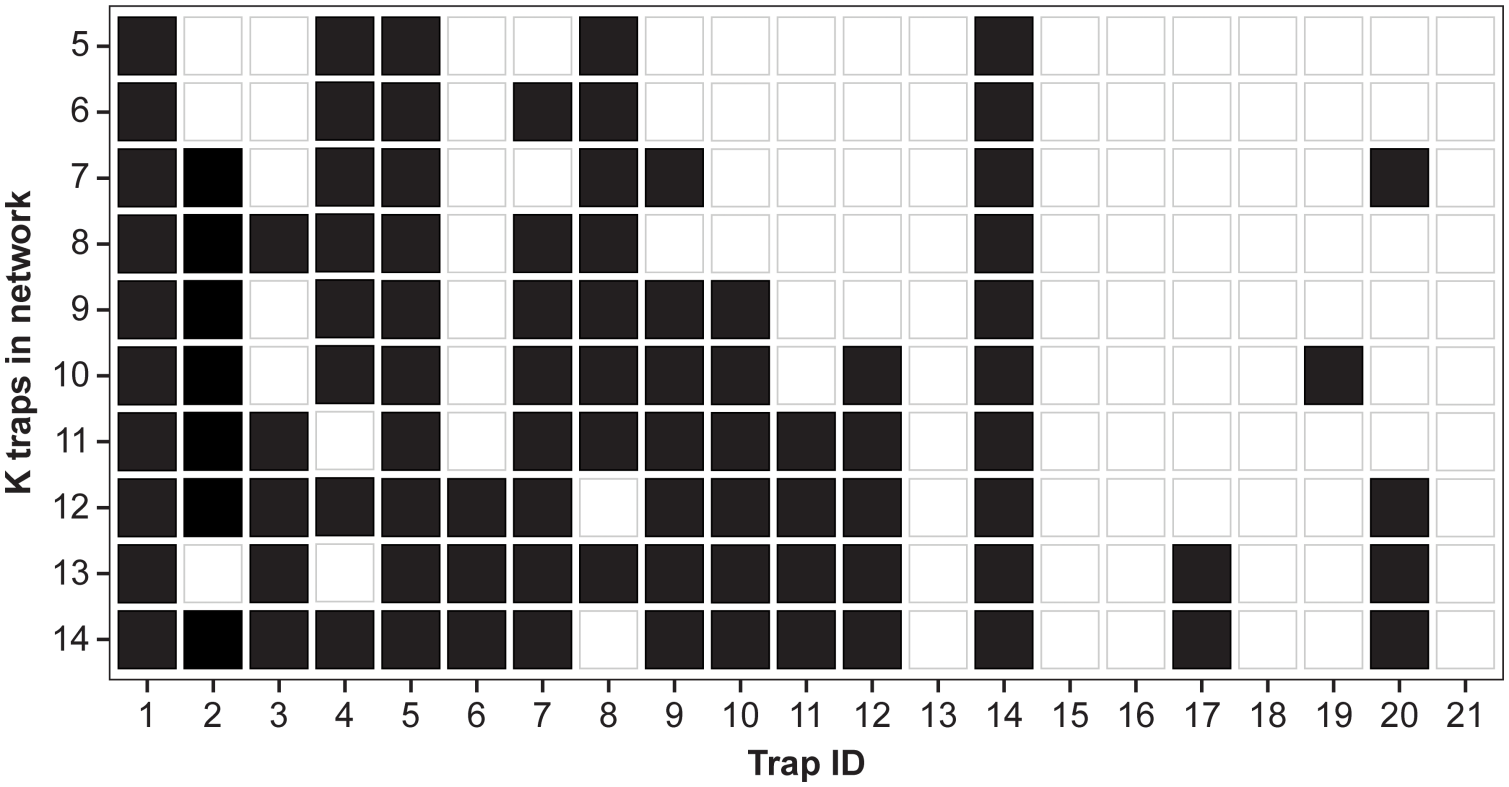
Inclusion/exclusion of traps in optimal set for each value of K . A black square indicates that trap was selected in the optimal set for the particular value of K . In case of tie, both sites are included.

While **Figures 4**, **5** show the proportion of MC samples for which a site was chosen in the optimal set, **Figure 6** considers the binary inclusion/exclusion of sites in the optimal set. For each value of K , the proportion of samples for which each site was chosen in the optimal set was calculated. Then the K sites with the largest proportion are chosen as the optimal sites. This is represented in **Figure 6** as a black tile if the trap has one of the K largest proportions, and a white tile otherwise. In case of ties, both sites have a black tile. Traps 1, 5 and 14 are included in the optimal site for every value of K . Conversely, traps 13, 15, 16, 18, and 21 are never selected. The plot also shows a substantial amount of nesting in the optimal sites, i.e., trap *i* is selected in the optimal set for K , then it is also selected in the optimal set for K+1 .

### 3.2 Model extension

As an extension of this work, we could explicitly include money, time, distance, etc. into our loss function if we wanted these to factor into our optimal choice (e.g., 9). For example,


$$L\left(S_{i}\right)=\frac{1}{11\left(L-K\right)}\sum_{s\notin S_{i}}\sum_{x=1}^{11}log\left(\left|Y_{\tau sx}-{\hat{Y}}_{\tau sx}\left(S_{i}\right)\right|+1\right)+\lambda C\left(S_{i}\right)\;$$

where $C\left(S_{i}\right)$ is the cost to sample at locations $S_{i}$ and λ is a tuning parameter which controls how much we weigh fit (small λ ) versus cost (large λ ) in the loss function.

### 3.3 Model performance and interpretation

The general implication of these empirical findings is that optimization enriches the information provided per unit trap in a multi-site network. First, the magnitude of the MLE associated with an optimized set is appreciably smaller than that for random sampling. For example, a MLE of 1.2 means that the predicted count was within 230 units of the true cumulative count value, on average. In the **Supplementary Materials**, we also compare the results of the BO algorithm with a brute-force computation for K=5 and find that the BO results are close to the global optimum with similar trap combinations detected by each method (**Supplementary Table S1**). This establishes the utility of the method for efficiently selecting trapping locations.

The BO algorithm was run 50 times and the average MLE was recorded. Ideally, the algorithm would be run once and the global optimum would be found, such that running multiple MC iterations would be unnecessary. The necessity of running the BO algorithm multiple times could be because the algorithm is finding a local optimum and/or because the objective function surface is relatively “flat.” This would mean that several different sets of K trap locations yield comparable MLEs. While these considerations and the computational expense of running the BO algorithm multiple times are relevant, running the algorithm multiple times also has advantages. The proportion of MC samples that a trap is included in the optimal set yields a continuous measure of importance as opposed to a binary result. This allows for some uncertainty quantification in the optimal sites and a richer understanding of which sites to choose for maximum precision in pest density estimation, and perhaps which sites to choose for research to understand why they are inconsistent or otherwise different from the optimal set.

In general, the optimal trapping locations appear in the southern and central locations. This could be interpreted one of two ways. It could mean that knowing the number of *H. zea* at these locations is very informative for knowing the number of moths at other locations. On the other hand, it could mean that these locations are very difficult to predict so we should trap at these sites to ensure an accurate value. For example, trap 1 could be included in every optimal set because knowing its value helps predict at the other sites, or because predicting the number of *H. zea* at trap 1 is extremely difficult. In the **Supplementary Materials**, we looked for systematic reasons why some trapping locations were chosen more than others. We found a moderate relationship showing that traps with a high probability of being included in the optimal set tended to have larger amounts of corn and soy planted within a 1 km radius. These are initial findings, however, and require further study.

Together, these steps toward optimization are important, as the costs of monitoring *H. zea* are immediately clear. Establishing which traps in the network provide the most return on investment would be a significant step toward sustainable monitoring approaches that continue to provide useful information to agricultural stakeholders. In the future, this approach would also apply to invasive species monitoring, which is a common approach when outbreaks occur. The persistent threat of pests with similar ecology as *H. zea* (i.e., *Helicoverpa armigera*) is a major focus of regulatory agencies tasked with responding to incipient threats of invasive species in the United States (e.g., USDA-APHIS). Ecological niche modeling suggests that *H. armigera* could establish in the southern U.S. and would pose a significant threat to agriculture across broad geographies (21). Effective monitoring strategies would be a first step toward effective management of a widespread invasive outbreak. The BO approach is one tool which could be adapted to model effective monitoring networks that are informed by the current distribution and activity of a closely related species.

### 3.4 Extending BO modeling to understand pest ecological drivers and sampling efficiency

These results suggest that understanding these contrasting motivating factors will help to assess which locations are informative and/or what about these locations make them informative. Future studies focused on assessing location-wise characteristics from the perspective of *H. zea* could generate new context for the optimized dataset. For example, overwintering temperatures limit *H. zea* survival during the pupal stage (22) which contributes to regional trends in population abundance over time (16). Moreover, soil conditions which vary at much smaller spatial scales are also linked to overwintering survival which affects *H. zea* population size in the spring (23, 24). During the growing season, the abundance of highly suitable host plants for *H. zea* development across multiple generations may also explain variation among traps across the relatively small geographic region where we established our trap network (12–14, 18). Although these studies independently assessed specific aspects of *H. zea* ecology, they are unable to account for multiple variables influencing these insects in agricultural systems. Absent an optimized network, it is difficult to learn what factors at key trap sites are responsible for those sites’ providing information that can positively affect grower management decisions. Next steps connecting optimized networks to relevant ecological and environmental drivers would further refine our understanding about *H. zea* population dynamics in the context of realistic agroecosystems. In short, the improvement of observational efficiency results in the improvement of any study or application that relies on precision estimates of the observed process. Optimizing the provision of evidence used to describe biological systems, to make system management decisions, or to discover novel or incipient events in those system is a valuable step to take early in a study or management program.

This study builds on a deep body of literature focused on improving sampling optimization for insect pests in agriculture. Entomologists apply numerous sampling techniques for the purpose of estimating arthropod population densities, sometimes adapting from methods used in engineering (25) or analysis of data from clinical trials (26, 27). A commonly applied sampling technique in entomological pest management is sequential sampling, first developed in the 1940’s (28), for the purpose of increasing the efficiency of estimation for values important to industrial processes. The primary motivation for the development of sequential sampling techniques was efficiency: the potential to accelerate estimation or increase precision per sample, while minimizing wasted sampling effort. In pest management, this translates to iterative sampling and interim analysis based on observations. Samplers use pre-established stopping conditions to determine when sufficient pest density or crop damage information has been collected to make a management decision. This level of tolerated pest activity is informed by the economic thresholds, which is a pest density or other damage metric above which *not* managing the pest is expected to result in net economic loss. For example, the threshold at which the value of the expected yield loss to *H. zea* larval herbivory exceeds the cost of management. Effectively, this effort becomes a statistical hypothesis, and sequential sampling proceeds until the hypothesis is rejected or fails to be rejected under pre-established tolerances for Type I and Type II error rates. While some important developments have extended the rationale of increasing sampling efficiency for decision support (e.g. 25, 29–31, 32), efficiency gains through innovative statistical approaches continue to represent a major opportunity for improving the environmental compatibility and economy of agricultural pest management. The BO algorithm developed in this study enhances sampling in a way parallel to existing practices of sequential sampling or interim analysis, but adds a substantial amount of value to data in the process by showing the aforementioned nesting of high-value sites within larger optimal sets, by providing a measure of uncertainty around the importance of individual sites, and by including correlation structure that allows nonrandom associations between sites to be exploited for optimization. Similar approaches could be developed for other pest species and geographic regions but will require initial investment to collect activity data for model development and validation.

## 4 Conclusions and future directions

In this study, BO provided useful insight into the optimization of a trap network targeting a key agricultural pest. Our results showed that we can achieve *H. zea* monitoring objectives without sampling at every trapping location. Moreover, the BO approach greatly outperforms the baseline method of random sampling. These empirical results provide several key takeaways for practitioners and stakeholders. First, if budgets limit the number of traps in a study area, then our BO procedure yields the optimal locations for these traps, while initial monitoring still may be required to validate the experimental set-up. Second, our methodology can also suggest how many traps to use, as in prospective power analysis. For example, if there is some pre-determined error tolerance or pre-specified desired coverage level, then the BO procedure can be used, not only choose the locations of the traps, but also how many to deploy. Indeed, the results in **Figure 3** show a clear monotonically decreasing error as the number of traps increases, so this must be chosen carefully. Being able to afford just one more trap in the best location may lead to high payoff. Collecting data from all 21 traps in the existing network currently requires a ~700 km trip for two entomologists for 10 weeks. Because time and distance are considerable, we also discussed an extension to the BO analysis that includes flexibility to optimize networks based on user-defined criteria. Finally, once the number and location of the traps are chosen, further analysis can be performed to understand *why* these sites were selected. This question is slightly beyond the scope of this work, so we only took a first step toward this end looking at landscape information which aligns with previous work suggesting that the abundance of soybean in the landscape is an important predictor of *H. zea* abundance in this region (33). The proposed statistics framework, however, facilitates principled study of this question.

## Data Availability

The datasets presented in this article are not readily available because the information includes specific location information. The raw data supporting the conclusions of this article will be made available by the authors upon reasonable request. Requests to access the datasets should be directed to ashuseth@ncsu.edu.
