# Supplementary material for: Bayesian Optimization of insect trap distribution for pest monitoring efficiency in agroecosystems
Source: Front Insect Sci. 2025 Jan 22;4:1509942. doi: 10.3389/finsc.2024.1509942 (PMC11794318; doi:10.3389/finsc.2024.1509942)
Supplement: Supplementary file 1 [file DataSheet1.pdf]

Supplemental Information

Bayesian Optimization of Insect Trap Distribution for Pest Monitoring Efficiency in Agroecosystems

Eric Yanchenko[1*], Thomas M. Chappell[2], Anders S. Huseth[3*]

[1]Global Connectivity Program, Akita International University, Akita, Japan

[2]Department of Plant Pathology and Microbiology, Texas A&M University, College Station, TX, USA

[3]Department of Entomology and Plant Pathology and North Carolina Plant Science Initiative, North Carolina State University, Raleigh, NC, USA

*** Correspondence:** Eric Yanchenko eyanchenko@aiu.ac.jp; Anders Huseth ashuseth@ncsu.edu

Supplemental Materials

Full Model and Priors

We report the full model used, including the prior distributions.

$$Y_{tsx} \sim Normal(\mu(x;s), \sigma^2), \quad (2)$$

$$\mu(x;s) = \frac{\beta(s)}{1 + exp[-\{x - \gamma(s)\}/k]}$$

$$\beta(s_1), \ldots, \beta(s_L) \sim Normal(0, {\sigma_\beta}^2 \Sigma_\beta)$$
$$\gamma(s_1), \ldots, \gamma(s_L) \sim Normal(0, {\sigma_\gamma}^2 \Sigma_\gamma)$$
$${\sigma_\beta}^2, {\sigma_\gamma}^2, \sigma^2 \sim InvGamma(0.01, 0.01)$$
$$\rho_\beta, \rho_\gamma \sim Uniform(0,2)$$

Choosing the value of B

To tune the value of B in the BO algorithm, we set $N_0 = 50, K = 5$ and $B = 100$. We then recorded the value of the loss function in Eq. (5) for each iteration and plotted it below. We can see that the MLE of the optimal solution is decreasing until roughly $B = 25$, at which point it stabilizes. This helps guide our choice of B.

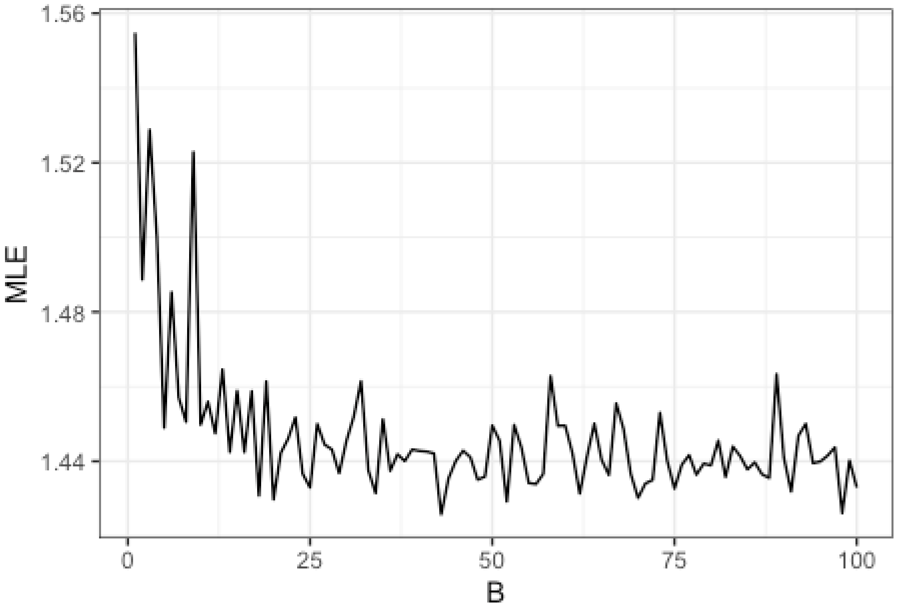

Figure S1. Progress curve of objective function in Eq. (5) for $N_0 = 50, K = 5$.

Brute force comparaison

To see how close the BO algorithm approximates the global optimum, we also ran a brute-force calculation for $K = 5$. We found the MLE on the 2023 hold-out data for all 21 choose 5 = 20, 349 combinations and identified the minimum value. The results are in Table S1 below. We can see that the BO algorithm correctly identified three of the optimal sites (traps 1, 5, 10) and the global minimum MLE was only 11% smaller than the BO algorithm's minimum MLE. Indeed, only 1% of all possible trap combinations had a smaller MLE than that of the optimal set chosen by BO.

Table S1. Comparison of brute force optimization with Bayesian optimization

Method	MLE	Optimal traps
Brute-force	1.08	1, 5, 9, 10, 16
Bayesian Optimization	1.22	1, 5, 6, 10, 11

Loss function comparison

In the main manuscript, we computed the mean log error between the predicted and observed values. This was chosen because it puts the error for each site on roughly the same level so each observation contributes similarly to the loss function. We could have also chosen to compute the mean squared error (MSE) and relative error (RE) as our loss function. The MSE is defined as

$$\sqrt{\frac{1}{n}\sum_{i=1}^{n}(\hat{Y}_i - Y_i)^2}$$

and the RE is

$$\frac{1}{n}\sum_{i=1}^{n}\frac{|\hat{Y_i} - Y_i|}{Y_i}$$

The loss functions in Eq. (3) and Eq. (5) are then defined analogously. Note that the MSE computes the magnitude of the error so it will favor locations with large observations. Conversely, the RE weights towards small observations since the division is by the observed value, so the summand will be large when the observation is small.

Landscape information

After running the analysis, we see which sites are chosen most often in the optimal set, and which sites are not. The natural question to ask is, why are certain sites chosen more/less?

First, we consider how location affects the number of times selected. In Figure 4, it appears that traps in the northern counties, particularly Northampton county, are selected less in the optimal set. Indeed, the correlation between proportion of iterations selected in the optimal set and latitude yields a correlation of -0.56. This indicates a moderately strong relationship that southern traps are chosen more often than those in the north.

Next, we look at the effect of landscape data on the propensity of a trap being selected. For each site and each year, we take a 1 km radius circle around the trap and compute the proportion of this area which is planted with (i) corn, (ii) soy, (iii) cotton, or (iv) other agricultural products. We average these proportions over the years 2020-2022. We only look at 2020-2022 because these are the only years the model uses to choose sites (2023 is used only for testing). Additionally, we average over the year's data because our model does not have a temporal component, meaning each year is considered equally. For each crop type, we compute the correlation between the crop proportion and proportion of samples that site was chosen in the optimal set. The results are in Table S2. We can see that as the proportion of land planted with corn and soy increases, so too does the likelihood that this site will be chosen in the optimal set, with correlation of 0.37 and 0.45, respectively.

Table S2. Correlation between proportion of surrounding area planted with each crop and proportion of times a site was chosen in the optimal seed set.

Crop	Correlation
Corn	0.37
Soy	0.45
Cotton	-0.24
Other ag.	-0.09
